# A Happy New Year

**Published:** 1913-01

**Authors:** 


### BUFFALO MEDICAL JOURNAL

### A Happy New Year

The BUFFALO MEDICAL JOURNAL extends its greetings to its readers; thanks them for their cordial support in the past and looks forward to still greater usefulness as the details of cooperation to secure professional welfare, are worked out. A New Year message without good resolutions would be like bricks without straw so here are presented a few with the hope that they are not needed.

I will pay my debts promptly; have as few as possible; and, so far as possible without working hardship, encourage others in the same resolution.

I will neither pay nor accept commisssions for the reference of cases but I will take this means of increasing my practice in preference to more under-handed methods not at present in the lime light.

I will keep the Hippocratic oath, not only in the narrow sense in which it is sometimes interpreted, but in the broad spirit that I will not exploit for selfish ends any advantage which my professional work affords in my dealings with other physicians, others professions, employees or patients of either sex or any age.

Without being a sore-head or a knocker and with due regard to the legitimate rewards of merit and service, I will use my influence to secure the fair and impartial administration of all professional trusts in the welfare of the profession and the people generally.

I will not step on the fingers of the man below me on the ladder. As to the man above me, I will not incur the curse of Deuteronomy, 25:11.

